# The Potential of Exerkines in Women’s COVID-19: A New Idea for a Better and More Accurate Understanding of the Mechanisms behind Physical Exercise

**DOI:** 10.3390/ijerph192315645

**Published:** 2022-11-24

**Authors:** Katsuhiko Suzuki, Amir Hossein Ahmadi Hekmatikar, Shadi Jalalian, Shaghayegh Abbasi, Elmira Ahmadi, Abdolreza Kazemi, Ruheea Taskin Ruhee, Kayvan Khoramipour

**Affiliations:** 1 Faculty of Sport Sciences, Waseda University, Tokorozawa 359-1192, Japan; 2 Department of Physical Education and Sport Sciences, Faculty of Humanities, Tarbiat Modares University, Tehran 10600, Iran; 3 Department of Physical Education and Sport Sciences, Science and Research Branch, Islamic Azad University, Tehran 10600, Iran; 4 Department of Exercise Physiology, Faculty of Physical Education and Sport Sciences, Kharazmi University, Tehran 10600, Iran; 5 Department of Sports Science, Faculty of Literature and Humanities, Vali-e-Asr University, Rafsanjan 7718897111, Iran; 6 Future Innovation Institute, Waseda University, Shinjuku 162-0041, Japan; 7 Neuroscience Research Center, Institute of Neuropharmacology, Department of Physiology, Afzalipour School of Medicine, Kerman University of Medical Sciences, Kerman 7616914115, Iran

**Keywords:** coronavirus disease 2019 (COVID-19), exerkines, females, women health, physical exercise, physical activity, SARS-CoV-2 virus, resistance training, aerobic exercise, Omicron

## Abstract

The benefits of physical exercise are well-known, but there are still many questions regarding COVID-19. Chow et al.’s 2022 study, titled Exerkines and Disease, showed that a special focus on exerkines can help to better understand the underlying mechanisms of physical exercise and disease. Exerkines are a group of promising molecules that may underlie the beneficial effects of physical exercise in diseases. The idea of exerkines is to understand the effects of physical exercise on diseases better. Exerkines have a high potential for the treatment of diseases and, considering that, there is still no study of the importance of exerkines on the most dangerous disease in the world in recent years, COVID-19. This raises the fundamental question of whether exerkines have the potential to manage COVID-19. Most of the studies focused on the general changes in physical exercise in patients with COVID-19, both during the illness and after discharge from the hospital, and did not investigate the basic differences. A unique look at the management of COVID-19 by exerkines, especially in obese and overweight women who experience high severity of COVID-19 and whose recovery period is long after discharge from the hospital, can help to understand the basic mechanisms. In this review, we explore the potential of exerkines in COVID-19 by practicing physical exercise to provide compelling practice recommendations with new insights.

## Key points

- With the prevalence of COVID-19 in the world as a dangerous disease, many studies have given physical exercise interventions, but the understanding of the effective and basic mechanisms of physical exercise is still unclear.
- The ACE2 receptor that the SARS-CoV-2 spike protein binds to is more expressed in fat tissue than in other organs, which is very high in obese and overweight women. Therefore, behavioral changes of adipokines in COVID-19 lead to high inflammation and, through crosstalk with other organs, such as muscle (myokines) or liver, hepatokines lead to a decrease in physical function and an increase in inflammation.
- Exerkines include myokines (muscle), cardiokines (heart), hepatokines (liver), neurokines (nervous system), and baptokines (brown adipose tissue) and adipokines (adipose tissue).
- Resistance training that includes weight training and exercise equipment can improve muscle strength and reduce inflammation by affecting myokines during COVID-19 disease. After discharge from the hospital, this physical exercise is suitable for returning to a quality of life. Moreover, aerobic exercise that includes moderate-intensity exercise (running on a treadmill or cycling) can have the greatest effect on the stimulation of exerkines.
- Exerkines have a high potential for treating diseases, but investigating their high potential for COVID-19 and the underlying mechanisms remained unanswered.
- The emerging research on exerkines opens up several promising avenues for future research where researchers can explore the potential importance of exerkines in elderly or coexisting COVID-19 patients.
- Future studies on exerkines can be very effective in designing accurate physical exercise and more practical recommendations for patients, especially COVID-19 patients, during illness or after discharge from the hospital.

## 1. Introduction

Coronavirus disease 2019 (COVID-19) is an infectious disease caused by the acute respiratory syndrome with coronavirus that primarily affects the lungs [1]. After three years of this disease, we understand who is more exposed to the symptoms of COVID-19 and who suffers from moderate and high severity of this disease [2]. The review of previous studies showed that most people infected with COVID-19 were obese or overweight [3,4], and disease severity and mortality were higher in obese subjects than in overweight subjects [5]. Moreover, during the illness, COVID-19 patients face a decrease in muscle strength (muscle atrophy), a decrease in muscle endurance, and other physiological factors, which can affect training programs in the long term after discharge from the hospital [6,7]. It has been determined that, in the long term, COVID-19 can lead to negative physiological changes and decrease the quality of life, reducing hope and motivation for physical activity, which worsens the course of the disease [8].

Gender is an essential determinant of mortality risk and immunological responses to COVID-19 [9,10,11]. Women face a higher risk of becoming infected during a pandemic because of their societal position, as the United Nations (UN) and the World Health Organization (WHO) reported [12,13,14]. Women are over-represented in health care professions [11,15,16,17]. On the other hand, obese and overweight women are more exposed to the complications of COVID-19 than normal women [18]. Being physically active or the lack thereof plays a vital role in obesity. Indubitably, obesity ensues from physical inactivity and vice versa [19,20,21]. Hormones can also affect sexual difference in viral infections such as COVID-19. The SARS-CoV-2 spike protein binds to the human angiotensin-converting enzyme 2 (ACE2) receptor. Studies have shown that estradiol, a primary female sex hormone, likely regulates ACE2 expression in airway epithelial cells, kidneys, heart, and adipose tissues [22,23]. In obese and overweight women, estradiol function [24] is disrupted and ACE2 expression increases [22,23].

Undeniable evidence has been supporting the vital role of physical exercise in improving immune system, body composition, and reducing the complications of COVID-19 [25,26]. Although the benefits of physical exercise in improving health and reducing the severity of COVID-19 are well known, the molecular mechanisms underlying these benefits associated with physical exercise are not yet defined and are being investigated [27,28]. Physical exercise generally includes aerobic, anaerobic, or resistance training, but physical activity includes occupational, sports, conditioning, household, or other activities [29,30,31]. Promoting physical activity seems to have a more significant effect on reducing the diseases severity [27,32,33,34]. In 2020, WHO reported that all adults should aim for 150 to 300 min of moderate-intensity physical activity per week or 75 to 150 min of vigorous-intensity physical activity per week, or an equivalent combination of moderate- and vigorous-intensity physical activity [35]. However, it is important to understand how physical activity can affect the acute or chronic immune response in obese women.

**Figure 1:**
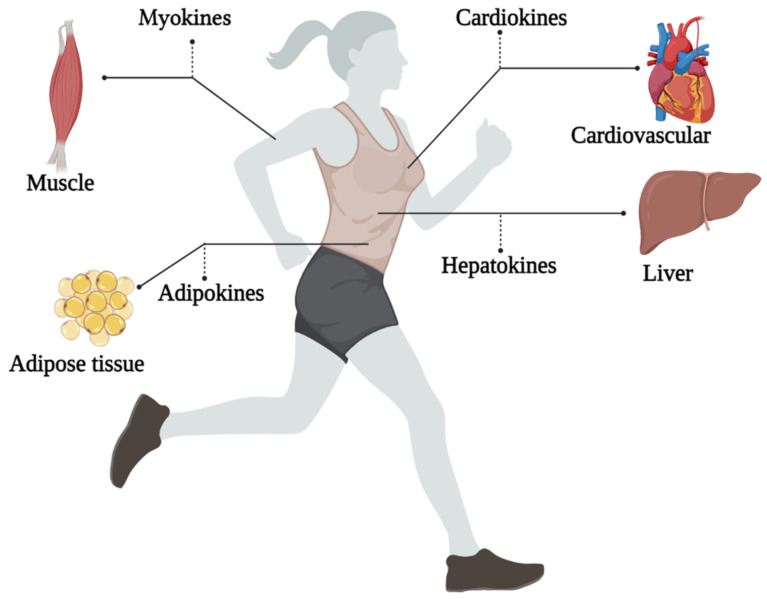
An overview of the exerkines investigated in this study.

To make better recommendations for immunization in women, we need to understand the impact of physical exercise on all organs. Studying exerkines would be the key. The word exerkines has introduced in 2016 [36]. So far, there is no exact definition of exerkines, but the best explanation for seems to be: the release of myokines, cardiokines, hepatokines, and adipokines due to physical exercise [27]. That is, physical exercise can affect these “kines” and ultimately exerts its effects through endocrine, paracrine, and/or autocrine pathways (Figure 1) [35]. The idea of the present study originated from the study of Chow et al., 2022, titled Exerkine and Diseases in the journal *Nature Reviews*. The intensity, duration, frequency, and volume of training are all factors that can play an important role in these responses. Understanding the role of exerkines in the physiological and biological response to physical exercise is a vital step to provide training suggestions to reduce the severity of COVID-19 in new strains. Therefore, we will discuss how the training variables could affect exerkine response.

**Figure 2:**
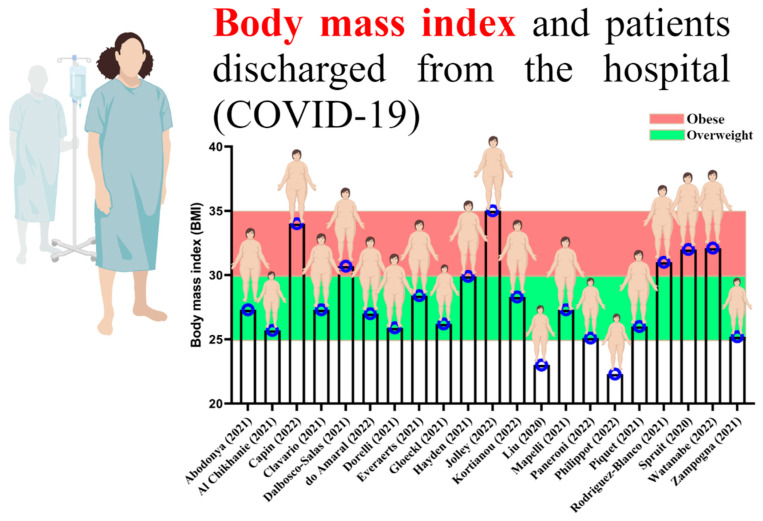
Body mass index of patients with COVID-19 discharged from the hospital [37,38,39,40,41,42,43,44,45,46,47,48,49,50,51,52,53,54,55,56].

By reviewing past studies related to rehabilitation and physical exercise in the survivors of COVID-19, it can be well understood that most of those who were infected with COVID-19 were overweight and obese (Figure 2) [37,38,39,40,41,42,43,44,45,46,47,48,49,50,51,52,53,54,55,56]. Obesity could affect the exerkine response to physical exercise, especially in women. Now that new strains of COVID-19 are on the way and obese women are experiencing a high severity of the disease, this study discusses the associations between gender, physical exercise, and exerkine secretion.

## 2. The Potential Role of Exerkines Can Be Effective in COVID-19

It is well known that the most affected organs in COVID-19 include fat tissue, lungs, muscles, liver, and heart [57]. With the binding of SARS-CoV-2 spike protein to ACE2, physiological changes are created in the tissue, which can increase the disease process and influence the severity of the disease [58]. Moreover, the side effects of COVID-19 can stay with the patient long after discharge from the hospital and reduce the quality of life [59]. In this regard, most sports studies implementing “rehabilitation exercises” after COVID-19 had a general view of the effect of rehabilitation exercises on patients discharged from the hospital and did not investigate the fundamental mechanisms but highlighted the importance of physical exercise. Nevertheless, to provide a suitable physical exercise strategy for this disease, the mechanisms behind the curtain should be investigated. The idea of exerkines to take a more detailed look at diseases in 2022 by Chow et al. in the journal *Nature Reviews Endocrinology* gave rise to the idea that it might be possible to investigate the mechanisms behind the curtain and the effect of physical exercise in COVID-19 [27].

Exerkines look at the effect of physical exercise acutely or chronically on disease [27], and frequency, intensity, time, and type (FITT) can help to understand the effect of exerkines on disease better [27]. Exerkines are secreted in response to acute exercise (short-term physical exercise and less than 2 weeks), which is usually a part of aerobic or resistance training. Chronic exercise (long-term physical exercise and more than 2 weeks) is associated with changes in humoral factors, even at rest, suggesting that exerkine changes may reflect the effects of chronic exercise [60]. The acute exerkine response is influenced by the type of physical exercise, duration of physical exercise, background fitness, feeding–fasting status, and post-exercise sampling time [61]. Classical exerkines released during acute exercise, as found in human and animal models, include IL-6, IL-8, IL-1 receptor antagonist (IL-1ra), and IL-10. In a human study in which blood samples were collected before and after a marathon race (acute exercise), plasma levels of several cytokines (IL-6, IL-1ra, IL-10, and tumor necrosis factor (TNF)), when collected immediately, were higher than the base. Plasma levels measured after physical exercise were found to peak and remain high for up to 4 h after exercise [62]. Notably, the acute exerkine response does not necessarily parallel the chronic exerkine response [63].

## 3. Can Exerkines (Generated/Stimulated by Physical Activity/Physical Exercise) Help COVID-19 Patients?

### Low-Intensity Physical Exercise

According to the American College of Sports Medicine, low-intensity training is recommended for people who are not fit or want to start exercising. Low-intensity exercise training may last 30 min or more [64]. Based on the study by Zinman et al., the desired intensity for this type of physical exercise is 20–40% of the maximum oxygen consumption (Vo_2max_), 35–45% of the maximum heart rate (HR_max_), and Borg scale of 2–3/10 [64]. Considering that COVID-19 patients discharged from the hospital cannot perform physical activities for more than 30 min, they can spend less than 20 min to resume exercises [52].

A study found low-intensity interval resistance training can lead to beneficial changes in myokines [65]. In this study, subjects performed resistance training for 12 weeks, with 40% 1RM and 15 to 20 repetitions [65]. However, low-intensity physical exercise of 20–40 Vo_2max_ or 35–45% of HR_max_, such as running, cycling, or walking, for less than five weeks appears not to affect myokines [30,31,32,34]. The low intensity and duration of the training week are effective in these positive changes. This improvement in low-intensity exercises has also been noted in cardiokines (i.e., IL-33, IL-6, IL-18, IL-1β, and follistatin) [66,67,68]. However, low-intensity exercise did not seem to have much of an effect on hepatokines and adipokines (i.e., melanoprotein, fetuin-A, leptin, adiponectin, chemrin, resistin, visfatin, omentin, FGF21, ANGPTL4, and follistatin) [69,70,71,72,73,74,75,76,77,78]. Considering that most people infected with COVID-19 were overweight and obese, this obesity is related to COVID-19 [79]. In obese and overweight people, the severity of COVID-19 is very high, and it seems that, with the reduction in body fat percentage, despite improving body composition, improvement in adipokines is also created. Low-intensity training cannot effectively improve the behavioral changes of adipokines and reduce body fat [80,81,82,83,84,85,86,87,88].

**Figure 3:**
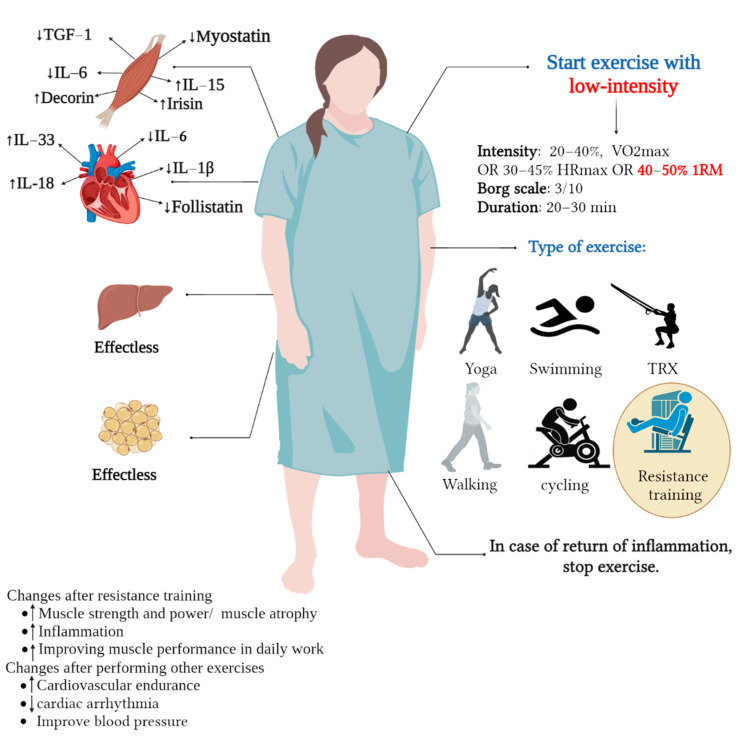
Effect of low-intensity exercise on exerkines in women with COVID-19 or discharged from hospital. TGF: transforming growth factor, IL: interleukin, RM: repetition maximum.

Thus, a low-intensity physical activity aimed at influencing exerkines appears appropriate for newly discharged or low-fit women. Of course, choosing the type of physical exercise is very important in influencing exerkines. Newly discharged women from the hospital (infected with COVID-19) need to restore strength [31]; so, it is well understood that low-intensity training can affect exerkines (myokines) more. Therefore, resistance training with a duration of 6 to 12 weeks and an intensity of 40% to 50% 1RM can be appropriate. Some studies have also suggested that exercises such as yoga, swimming, total body resistance exercise (TRX), walking, and cycling, as well as home exercises, such as body weight training, are also effective [89,90,91,92]. However, it seems that, in women who have just been discharged from the hospital, the duration should not be longer than 20 to 30 min to avoid re-inflammation. Therefore, in this short period of time, resistance training is suitable for women who have just been discharged from the hospital. Other means of physical exercise, such as cycling, running, or climbing, will be suitable for women with low physical fitness levels (see Figure 3 and Table 1).

## 4. Can Exerkines (Generated/Stimulated by Physical Activity/Physical Exercise) Help Newly Discharged COVID-19 Patients?

### 4.1. Moderate-Intensity Physical Exercise

Moderate-intensity exercise is an exercise that may last more than 30 min, with an intensity of between 40% and 60% of the Vo_2max_, 55% and 70% of the HR_max_, and Borg scale of 4–6/10 [64]. With the spread of COVID-19, many studies reported the importance of moderate-intensity exercise to reduce the severity of COVID-19 [34,146,147,148,149]. The common denominator of these studies was that their physical exercise recommendations for reducing the severity of COVID-19 seem to be the same or close to the same [34,146,147,148,149]. Moderate-intensity exercise has an intensity of between 40 and 65% Vo_2max_ or 50 and 75% HR_max_ and Borg scale of 4–6/10 [34,64,146,147,148,149]. However, how moderate-intensity training affects exerkines can be interesting but controversial.

Regarding myokines, it seems that one of the best effects of moderate-intensity training is to increase apelin [150,151,152]. Apelin can stimulate muscle mitochondrial biogenesis and protein synthesis and enhance muscle stem cells to promote muscle regeneration [152]. An interesting study found that aerobic exercise can increase muscle hypertrophy in women [153]. It appears that moderate-intensity aerobic exercise can lead to an increase in follistatin and a decrease in myostatin activity [154,155]. Decreased myostatin function increases skeletal muscle growth and improves whole-body glycemic control [154,155]. However, the physical exercise that most affects muscle exerkines is resistance exercise [156]. It appears that eight weeks of moderate-intensity resistance training (40–60% 1RM) can lead to positive changes in myokines (increase IL-6, IL-8, follistatin, and IL-15 and decrease myostatin) [156]. The results of other studies also confirm that resistance training can have the best beneficial effect on myokines [157,158]. In another study, it was found that both aerobic exercise (running) and moderate-intensity resistance exercise can produce a positive change in myokines (increase IL-7 and IL-8 and decrease IL-6) [93]. However, the emphasis of studies for positive changes in myokines is resistance training. Moreover, 6 to 12 weeks seemed to be a good time for these positive changes [93,157,158].

These positive changes due to moderate-intensity aerobic exercise do not affect other exerkines (cardiokines, hepatokines, and adipokines) and have significant positive effects (increase IL-33, IL-3, IL-4, IL-7, IL-10, IL-12, follistatin, neurotrophins, selenoprotein, fetuin-A, leptin, adiponectin, chemerin, resistin, visfatin, and omentin and decrease IL-6 and IL-1β) [63,68,131,159,160,161]. Therefore, both resistance training (weights or bodybuilding machines) and aerobic training (running on a treadmill and walking) will have a significant effect on exerkines. However, we can also mention combined exercises that can be very effective, with positive effects on exerkines to reduce the severity of COVID-19 [93,162,163]. Despite the fact that combined training (resistance and aerobic) can play a significant role in the positive impact of exerkines, current evidence shows that choosing the wrong type of movement and timing can lead to training interference and a negative effect on exerkines [164,165]. It is suggested to perform resistance exercises in one session and aerobic exercises in a separate session so that the phenomenon of training interference does not occur [164]. However, combined exercises that include aerobic exercises (40% and 60% of the Vo_2max_, 55% and 70% of the HR_max_) and resistance exercises (40–60% 1RM with 10 to 15 repetitions) with a duration of 6 to 12 weeks can be effective in positively affecting exerkines. Thus, it is suggested that running on a treadmill, cycling, and resistance exercises are excellent choices for moderate-intensity exercise training [31,166].

**Figure 4:**
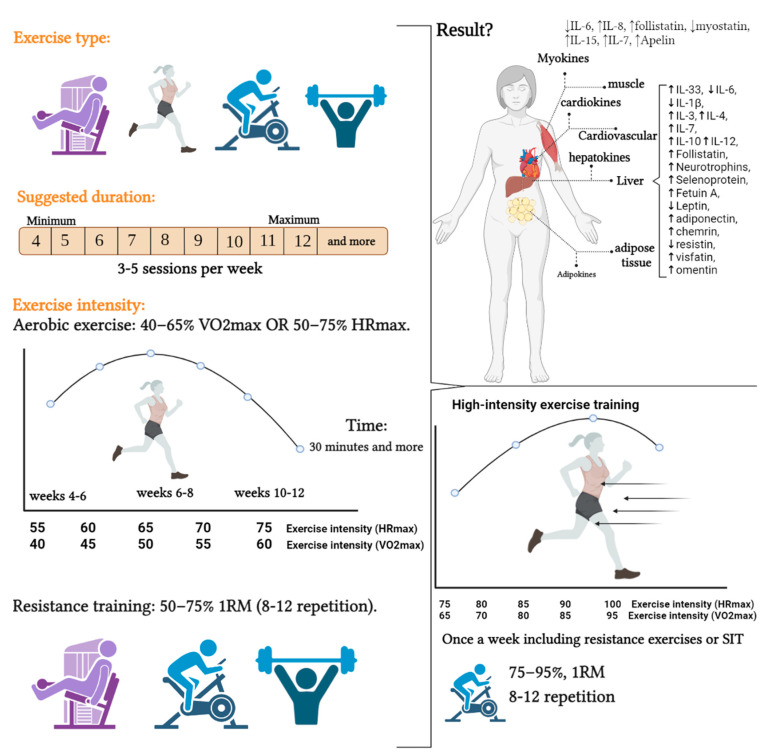
The effect of different type of exercise training on exerkines in women for immunization. SIT: sprint interval training, IL: interleukin, HRmax: heart rate maximum, Vo2max: maximum oxygen consumption.

Finally, moderate-intensity exercise may be the best approach to reduce disease severity in women with new strains of COVID-19. It is better to gradually enter moderate-intensity training after finishing the training period with low intensity. However, the type, duration, and intensity of physical exercise are very important. It is better to start with resistance exercises (exercises with weights and bodybuilding machines) with moderate intensity, and then use combined exercises in the following weeks. This can be especially important in women who have just been discharged from the hospital (see Figure 4 and Table 1).

### 4.2. High-Intensity Physical Exercise

It is well understood that, when high-intensity exercise occurs, these exercises can lead to an open-window phenomenon that ultimately suppresses the body’s immune system [167]. High-intensity exercise training leads to immune system dysfunction, inflammation, oxidative stress, and muscle damage and can endanger a person’s health. Markers of immune system function decrease from a few hours to a few days after a high-intensity exercise [165,168]. However, this response could vary among healthy, sick, trained, and untrained individuals [169]. High-intensity exercise does not appear appropriate for women recently discharged from the hospital due to COVID-19 or who are not physically fit. However, investigating the role of high-intensity training on exerkines can open a new process of training recommendations for women.

Previously reported data showed that high-intensity interval training with a short duration (1 session to 1 week) has no effect on myokines (IL-6, IL-8, follistatin, myostatin, and IL-15) [170]. However, increasing the duration of training between 4 and 8 weeks led to a positive change in myokines [156,171]. Moreover, these positive changes are seen with high-intensity exercises in cardiokines, hepatokines, and adipokines [68,172,173,174]. However, maneuvering on high-intensity interval training and exerkines cannot be a suitable strategy to reduce the severity of the disease of COVID-19. Since most people who suffer from the severe COVID-19 are obese and overweight, high-intensity exercise is not a suitable strategy for them. Therefore, high-intensity interval training can be introduced once a week as a shock now (exercise with an intensity of 75% to 90% of HR_max_ and 65 to 95% of Vo_2max_) (see Figure 4 and Table 1).

## 5. Discussion

Although exercise’s health and disease benefits are well known, our understanding of the underlying mechanisms is still rudimentary. However, studies in the field of exerkines are expanding to give us a better experience of this pathway. Exerkines are increasingly recognized as critical mediators of exercise-related changes and health benefits, particularly in their role in interorganismic and systemic communication and co-ordination. However, many research gaps related to diseases can still be filled by examining exerkines. As COVID-19 spreads worldwide, many studies have been published, all of which report the effects of physical exercise alone on muscles and quality of life after hospital discharge [37,38,39,40,41,42,43,44,45,46,47,48,49,50,51,52,53,54,55,56]. However, published studies have generally reported muscle strength after rehabilitation exercises and have not looked specifically at the underlying mechanisms affecting muscle or other organs [37,38,39,40,41,42,43,44,45,46,47,48,49,50,51,52,53,54,55,56]. For this purpose, a review study was conducted to investigate the importance of exerkines in COVID-19 patients discharged from the hospital.

### 5.1. Findings on Exerkines and Women COVID-19 Patients

During the illness, COVID-19 patients experience decreased muscle strength, increased blood pressure, fatigue, and decreased performance. In more severe cases, it can lead to death. Women who are obese and overweight suffer from high severity of the disease. The mechanism of why obese people suffer from this disease more than thin people is unknown. Nevertheless, recent studies have shown that obese people express more ACE2 receptors on adipose tissue than lean people. This can result in more binding of SARS-CoV-2 spike protein to its receptor. Several studies have shown that COVID-19 in obese people can lead to significant behavioral changes in adipokines (increase in leptin, decrease in adiponectin, increase in resistin, and decrease in omentin). These behavioral changes can lead to increased inflammation in the body. Increased inflammation in adipose tissue can affect other organs, such as muscle, and lead to muscle atrophy or decreased strength.

The current review study investigated the importance of exerkines during illness (Figure 3 and Table 1). In this section, we examined various physical exercise and physical activity interventions on exerkines to determine the importance of exerkines in COVID-19 patients. Resistance exercises with low intensity seem to affect myokines’ positive changes (Figure 3 and Table 1). In this regard, it seems that resistance training with low intensity also affects cardiokines (Figure 3 and Table 1). However, low-intensity training did not significantly affect other exerkines, i.e., hepatokines and adipokines. In this section, we also reviewed other physical exercise interventions that do not seem effective for the COVID-19 patient, focusing on exerkines. The reason for not affecting these exerkines can be due to the low intensity or short duration of the physical exercise intervention (maybe yoga exercises are only helpful for reducing stress). In this section, we were looking for the best physical exercise recommendation that can be effective for COVID-19 patients, and it seems that resistance training can be a suitable recommendation. So, it seems that resistance training can be performed with training bands, light weights, or hand resistance. However, we found out in this section that training during illness should be less than 20 min so as not to cause fatigue. Using the Borg scale, which can be in the range of 3 out of 10, effectively controls the intensity of the exercise. Therefore, it is recommended that low-intensity resistance training can be effective as a functional strategy in COVID-19 patients (Figure 3 and Table 1).

### 5.2. Findings on Exerkines and Women COVID-19 Patients (after Discharge from the Hospital)

In the second part, we discussed the importance of exerkines in patients with COVID-19 discharged from the hospital. Which intensity and which type of training can shorten the rehabilitation period and increase the effectiveness is very important. Because the new strains of COVID-19 (Omicron) are increasing, which can reduce the severity of the disease in the case of new strains, most of the published studies have reported the importance of moderate- to low-intensity rehabilitation exercises that included resistance and aerobic exercises on muscle strength, sleep quality, blood pressure, walking, and overall quality of life. However, these results report a general view of these changes after exercise, which cannot be a precise physical exercise recommendation. The idea of exerkines in this study focused on the target of basic mechanisms after hospital discharge.

In this section, it was found that moderate-intensity aerobic and resistance exercises can have the most significant effects on adipokines and myokines, respectively (Figure 4 and Table 1), and there were limited studies related to hepatokines and cardiokines. It seems that acute exercises with fewer than four weeks cannot be a good idea to affect the exerkines, and the most significant effect was seen between 4 and 12 weeks (Figure 4 and Table 1). Resistance training using exercise machines provides more safety than free weights. After reviewing the studies, we found that, in the aerobic exercise section, running, cycling, and swimming can have the greatest effect on the exerkines (Table 1). Therefore, it seems that the best training advice can be resistance training with gym equipment and aerobic training (running). Not that we report that other physical exercise interventions are ineffective, but their significance in exerkines is still lacking. Of course, yoga or TRX exercises were also investigated, which do not seem to affect exerkines.

### 5.3. Conclusions

The review of studies showed that resistance training (weight training or resistance device) with low to moderate intensity could increase muscle strength and endurance and reduce inflammation by changing myokines. However, at high intensity, an increase in inflammation was seen. It can be suitable for COVID-19 patients discharged from the hospital (to return to a quality of life). This kind of resistance training with low to moderate intensity can reduce blood pressure, reduce inflammation, improve the function of the body’s immune system, and increase metabolism by changing cardiokines, adipokines, and hepatokines. TRX exercises performed with body weight appear to be effective for patients with low to moderate severity of COVID-19. However, in relation to yoga exercises, no study was found that reported stimulation of exerkines. A review of studies found that low- to moderate-intensity swimming can lead to stimulation of exerkines, which can be appropriate after hospital discharge. However, low-intensity walking and moderate-intensity running could have the best stimulation on exerkines, leading to increased immune system function, reduced inflammation, improved strength, increased muscular and cardiovascular endurance, and increased metabolism. Moreover, cycling, like walking and running, had the best effect on stimulating exerkines.

### 5.4. Limitations and Prospects for Future Research

Exerkines are a very promising idea for future research initiatives and, with their high potential as biomarkers to predict outcomes and facilitate personalized physical exercise programs to improve health and reduce disease, they can fill many scientific gaps. In this study, we examined four important exerkines that could effectively manage COVID-19, but other exerkines could be considered in prospective studies. For example, more than 600 myokines have been discovered so far, but we considered six of them (8). One of the limitations of this study was that we only could examine four important exerkines, and we could not examine the other two, hepatokine and neurokine. Unfortunately, technical issues have limited the investigation of the effects of exerkines on the human brain; on the other hand, baptokines have not been specifically looked at. This can be a good recommendation for future research to see how these two exerkines change with different physical exercise interventions. The current study focused on women, and it is suggested to investigate the importance of exerkines in men, older adults, or people with underlying diseases or focus on COVID-19. By examining the idea of exerkines in this study, it was found that studies using rehabilitation exercises reported a general view of changes. It is suggested that future studies address the importance of exerkines in rehabilitation exercises related to patients with COVID-19 discharged from the hospital. Considering that it has recently been determined that most ACE2 receptor is expressed on fat tissue and adipokines cause many behavioral changes, these behavioral changes in adipose tissue seem to affect other exerkines, such as myokine and hepatokine. It was also found that most of the patients with COVID-19 were obese and overweight, but the researchers did not examine the changes in fat tissue exerkine. It is suggested that researchers should investigate the importance of exerkines after discharge from the hospital or during illness in future studies. One of the points not seen in these studies was the examination of combined exercises. In future studies, researchers should focus on the importance of combined exercises and exerkines with a focus on COVID-19. It is also suggested that researchers investigate cytokine and interleukin responses during postexercise COVID-19 disease in future studies. As discussed, nutrition can influence the effect of exercise on exerkines by improving immune function [175]. It is suggested to investigate the importance of nutrition in combination with exerkines in future studies.

## Figures and Tables

**Table 1:** The effect of different types and intensities of physical exercise (low, moderate, and high) on the stimulation of exerkines.

Physical Exercise Type (L/M/H)	Exerkines					
	Myokines	Cardiokines	Adipokines	Hepatokines	Outcome	Reference
Resistance training (Resistance weight training exercise with a resistance machine)	(L/M)	(L/M)	(L/M)	(L/M)	Resistance training with low to moderate intensity can reduce inflammation and increase muscle strength by changing myokines. This can be effective in COVID-19 patients who suffer from decreased muscle strength and high inflammation during the illness. Moreover, resistance training with these intensities can benefit patients discharged from the hospital. However, high-intensity resistance training cannot positively affect myokines, and the results of articles have reported an increase in inflammation and a decrease in muscle strength. The effect of resistance training on cardiokines with low to moderate intensity can be suitable for improving high blood pressure and reducing inflammation. However, high-intensity resistance training may not be good for COVID-19 patients or those discharged from the hospital. Although studies on cardiokines are very limited, they can increase inflammation and possibly increase blood pressure. Low to moderate resistance training has a good effect on adipokines and hepatokines and can reduce inflammation, improve immune system function, improve insulin sensitivity, and increase fat tissue metabolism. In connection with high-intensity resistance training, the increase in inflammation is seen because of the behavioral changes in fat tissue and the decrease in the body function’s immune system.	
	↓myostatin ↓TGF-β1 ↓IL-6 ↑IL-15 ↑decorin ↑irisin ↑BDNF ↑IL-15 ↑FGF ↔SPARC	↓ANP ↔BNP ↑IL-33 ↓IL-6 ↑IL-18 ↓IL-1β ↓Follistatin ↑FGF ↔Sfrp ↑Neurotrophins ↓TNF-α ↓TGF-β	↓Leptin ↑Adiponectin ↑Chemerin ↓Visfatin ↓Omentin ↑Vaspin ↔Progranulin ↔CTRP-4	↑Activin-E ↔ANGPTL3 ↔ANGPTL4 ↑ANGPTL6 ↑Fetuin-A ↑FGF21 ↓Follistatin ↑GDF15 ↓Hepassocin ↑IGF1 ↓LECT2 ↑Lipocalin 13 ↑Selenoprotein-P ↑Tsukushi		[78,93,94,95,96,97,98,99,100,101,102,103,104,105,106,107,108,109,110]
	H	H	H	H		
	↓myostatin ↓TGF-β1 ↑IL-6 ↑IL-15 ↑decorin ↑irisin ↑BDNF ↑IL-15 ↑FGF ↑SPARC	↔ANP ↔BNP ↔IL-33 ↔IL-6 ↑IL-18 ↓IL-1β ↓Follistatin ↑FGF ↔Sfrp ↑Neurotrophins ↓TNF-α ↓TGF-β	↔Leptin ↑↔Adiponectin ↑↓Chemerin ↓↔Visfatin ↓↑Omentin ↑↔Vaspin ↔↑Progranulin ↔↑CTRP-4	↓Activin-E ↑ANGPTL3 ↑ANGPTL4 ↓ANGPTL6 ↓Fetuin-A ↔FGF21 ↔Follistatin ↔GDF15 ↑Hepassocin ↔IGF1 ↔LECT2 ↓Lipocalin 13 ↔Selenoprotein-P ↔Tsukushi		
TRX	(L/M)	(L/M)	(L/M)	(L/M)	Low- to moderate-intensity TRX training can positively change some myokines, resulting in improved muscle strength and reduced inflammation. However, no studies were found in connection with high-intensity resistance training. Moreover, TRX training with low to moderate intensity can probably lead to a decrease in inflammation or an increase in metabolism and an improvement in the functioning of the immune system, with positive changes in adipokines; however, the results are limited and no study was found concerning high intensity.	[111,112,113,114,115]
	↓myostatin ↔TGF-β1 ↔IL-6 ↔IL-15 ↔decorin ↑irisin ↔BDNF ↔IL-15 ↑FGF ↔SPARC	N/A	↓Leptin ↑Adiponectin ↑Chemerin ↓Visfatin ↓Omentin ↑Vaspin ↔Progranulin ↔CTRP-4	N/A		
	H	H	H	H		
	N/A	N/A	Leptin? Adiponectin? Chemerin? Visfatin? Omentin? Vaspin? Progranulin? CTRP-4?	N/A		
Yoga	(L/M)	(L/M)	(L/M)	(L/M)	No study focusing on exerkines was found. However, studies showed that practicing yoga can lead to a decrease in inflammatory cytokines and an increase in anti-inflammatory cytokines. However, it is impossible to conclude whether yoga focusing on exerkines is suitable.	[116,117,118,119,120]
	N/A	N/A	N/A	N/A		
	H	H	H	H		
	N/A	N/A	N/A	N/A		
Swimming	(L)	(L)	(L/M)	(L)	Low-intensity swimming training seems not to affect myokines, but moderate to intense training shows positive changes that can reduce inflammation and increase muscle strength. Concerning adipokines, low- to moderate-intensity swimming training can cause positive changes in adipokines and reduce inflammation and improve immune system function. Nevertheless, it seems like this concerns low intensity. No study was found in connection with other exerkines, but some studies have reported improved cardiovascular function and improved metabolism.	[121,122,123,124,125,126,127,128,129,130]
	↔myostatin ↔TGF-β1 ↓IL-6 ↓IL-15 ↔decorin ↔irisin ↔BDNF ↔IL-15 ↔FGF ↔SPARC	N/A	↓Leptin ↑Adiponectin ↑Chemerin ↓Visfatin ↓Omentin ↑Vaspin ↔Progranulin ↔CTRP-4	N/A		
	(H/M)	(H/M)	(H)	(H/M)		
	↑myostatin ↓TGF-β1 ↓IL-6 ↓IL-15 ↑decorin ↑irisin ↑BDNF ↑IL-15 ↑FGF ↔SPARC	N/A	Leptin↑ Adiponectin↓Chemerin↑ Visfatin↑ Omentin↑ Vaspin↑ Progranulin↑ CTRP-4↑	N/A		
Walking OR running	(L/M)	(L/M)	(L/M)	(L/M)	Walking with low to moderate intensity (running) positively affects some myokines, reducing inflammation and, sometimes, increasing muscle hypertrophy. However, in intense running, negative changes in myokines can lead to increased inflammation. The association between cardiokines and low- to moderate-intensity walking suggests that changes in some cardiokines can lead to lower blood pressure and, in some cases, reduced inflammation. It also improves cardiovascular endurance. However, with high intensity, there are few results and, in some cases, an increase in inflammation is seen. In connection with walking with low to moderate intensity (running) and adipokines and hepatokines, it seems that the best results occur and significant changes are seen in adipokines and hepatokines, the results of which are reducing inflammation, improving the function of the immune system, increasing metabolism, and increasing insulin sensitivity.	[78,131,132,133,134,135,136,137,138,139,140,141,142]
	↑myostatin ↓TGF-β1 ↓IL-6 ↓IL-15 ↔decorin ↔irisin ↑BDNF ↑IL-15 ↔FGF ↔SPARC	↓ANP ↓BNP ↑IL-33 ↓IL-6 ↑IL-18 ↓IL-1β ↓Follistatin ↑FGF ↓Sfrp ↑Neurotrophins ↓TNF-α ↓TGF-β	↓Leptin ↑Adiponectin ↑Chemerin ↓Visfatin ↓Omentin ↑Vaspin ↔Progranulin ↔CTRP-4	↑Activin-E ↔ANGPTL3 ↔ANGPTL4 ↑ANGPTL6 ↑Fetuin-A ↑FGF21 ↓Follistatin ↑GDF15 ↓Hepassocin ↑IGF1 ↓LECT2 ↑Lipocalin 13 ↑Selenoprotein-P ↑Tsukushi		
	H	H	H	H		
	↓myostatin ↑TGF-β1 ↑IL-6 ↑IL-15 ↓decorin ↓irisin ↔BDNF ↓IL-15 ↓FGF ↔SPARC	↔? ANP ↔? BNP ↓IL-33 ↑IL-6 ↓IL-18 ↔↑IL-1β ↔Follistatin ↔FGF ↓Sfrp ↑Neurotrophins ↑TNF-α ↑TGF-β	Leptin↑ Adiponectin↓ Chemerin↑ Visfatin↑ Omentin↑ Vaspin↑ Progranulin↑ CTRP-4↑	↓Activin-E ↑ANGPTL3 ↑ANGPTL4 ↓ANGPTL6 ↓Fetuin-A ↓FGF21 ↑Follistatin ↔GDF15 ↑Hepassocin ↓IGF1 ↓LECT2 ↓Lipocalin 13 ↔Selenoprotein-P ↔Tsukushi		
Cycling	(L/M)	(L/M)	(L/M)	(L/M)	Cycling with low to moderate intensity can increase the strength of lower body muscles and reduce inflammation by changing myokines, which cannot be seen at high intensity. Moreover, low- to moderate-intensity cycling can lead to increased muscular endurance, cardiovascular endurance, blood pressure, and cardiac output. These results were not seen at high intensity and increased inflammation occurs at high intensity. Low- to moderate-intensity cycling focusing on adipokines can reduce inflammation and improve immune function. No study was found in connection with high intensity and hepatokines changes.	[99,135,143,144,145]
	↓myostatin ↔TGF-β1 ↓IL-6 ↓IL-15 ↑decorin ↑irisin ↑BDNF ↑IL-15 ↑FGF ↑SPARC	↓ANP ↓BNP ↑IL-33 ↓IL-6 ↑IL-18 ↓IL-1β ↓Follistatin ↑FGF ↓Sfrp ↑Neurotrophins ↓TNF-α ↓TGF-β	↓Leptin ↑Adiponectin ↑Chemerin ↓Visfatin ↓Omentin ↑Vaspin ↔Progranulin ↔CTRP-4	N/A		
	H	H	H	H		
	↓myostatin ↑TGF-β1 ↑IL-6 ↑IL-15 ↓decorin ↓irisin ↔BDNF ↓IL-15 ↓FGF ↔SPARC	↔? ANP ↔? BNP ↓IL-33 ↑IL-6 ↓IL-18 ↔↑IL-1β ↔Follistatin ↔FGF ↓Sfrp ↑Neurotrophins ↑TNF-α ↑TGF-β	N/A	N/A		

L: low intensity, M: moderate intensity, H: high intensity, TGF: transforming growth factor, IL: interleukin, BDNF: brain-derived neurotrophic factor, FGF: fibroblast growth factor, SPARC: secreted protein and rich in cysteine, ANP: atrial natriuretic peptide, BNP: brain natriuretic peptide, Sfrp: secreted frizzled-related proteins, TNF: tumor necrosis factor, ANGPTL3-4-6: angiopoietin-like protein 3-4-6, GDF: growth differentiation factor, IGF: insulin-like growth factor, LECT: leukocyte cell-derived chemotaxin, CTRP: cancer therapeutics response portal, N/A: unreported or little irrelevant research, ↑: increase, ↓ decrease, ↔: unchanged or unknown, ?: not defined.

## Data Availability

Data sharing is not applicable to this article as no new data were created or analyzed in this study.
